# Supplementary material for: Unimproved source of drinking water and its associated factors: Multivariable analysis of Somalia Integrated Household Budget Survey (SIHBS 2022)
Source: PLoS One. 2025 Oct 15;20(10):e0334606. doi: 10.1371/journal.pone.0334606 (PMC12527177; doi:10.1371/journal.pone.0334606)
Supplement: S1 — (PDF) [file pone.0334606.s001.pdf]

Response to Copyright Inquiry for Figure 1 – PONE-D-25-33507R1

Dear Khem Descatamiento,

Thank you for your email and for the detailed guidance regarding Figure 1 in our submission.

We would like to clarify that the figure in question was **fully generated by our team using Python code**. The only external data used was a **free administrative boundaries shapefile** from GADM, which is publicly available for use. We used this shapefile solely to map the Somalia regions and then overlaid our own calculated results.

Since the figure was **created entirely by us** and does not use copyrighted maps, satellite images, or proprietary software, there is **no requirement to obtain additional permissions** for publication under the CC BY 4.0 license.

We hope this clarifies the matter. Please let us know if any further information or documentation is needed to proceed.

Please find attached Python code to this letter.

Thank you for your consideration.

Kind regards,
Dr. Omran Salih

In [44]:

```
import pandas as pd
import geopandas as gpd
import matplotlib.pyplot as plt

# 1️⃣ Your data
data = {
    "Region": ["Awdal","Bakool","Banaadir","Bari","Bay","Galguduud","Gedo",
               "Hiiraan","Jubbada Hoose","Shabeellaha Hoose","Woqooyi Galbee
               "Shabeellaha Dhexe","Mudug","Nugaal","Sanaag","Sool","Togdhee
    "Unimproved_Water": [10.10,43.28,0.00,9.53,11.90,6.01,46.90,10.56,7.73,
                         0.88,42.14,6.47,5.03,8.62,46.51,43.22,30.82]
}
df = pd.DataFrame(data)

# 2️⃣ Load Somalia shapefile
somalia_map = gpd.read_file("gadm41_SOM_shp/gadm41_SOM_1.shp")

# 3️⃣ Merge with your data
map_df = somalia_map.merge(df, left_on="NAME_1", right_on="Region")

# 4️⃣ Plot choropleth
fig, ax = plt.subplots(1, 1, figsize=(14, 12))

map_df.plot(column="Unimproved_Water",
            cmap="Reds",
            linewidth=0.8,
            ax=ax,
            edgecolor="0.8",
            legend=True)

ax.set_title("Distribution of Unimproved Water (%) in Somalia by Region",
             fontdict={'fontsize':16}, pad=20)
ax.axis('off')

# 5️⃣ Add labels with region name + percentage
for idx, row in map_df.iterrows():
    ax.annotate(text=f"{row['Region']}\n{row['Unimproved_Water']}%",
                xy=(row['geometry'].centroid.x, row['geometry'].centroid.y),
                horizontalalignment='center',
                fontsize=9,
                fontweight='bold',
                color='black')

# 6️⃣ Save map as image
plt.savefig("somalia_unimproved_water_map.png", dpi=300, bbox_inches='tight'

plt.show()

```

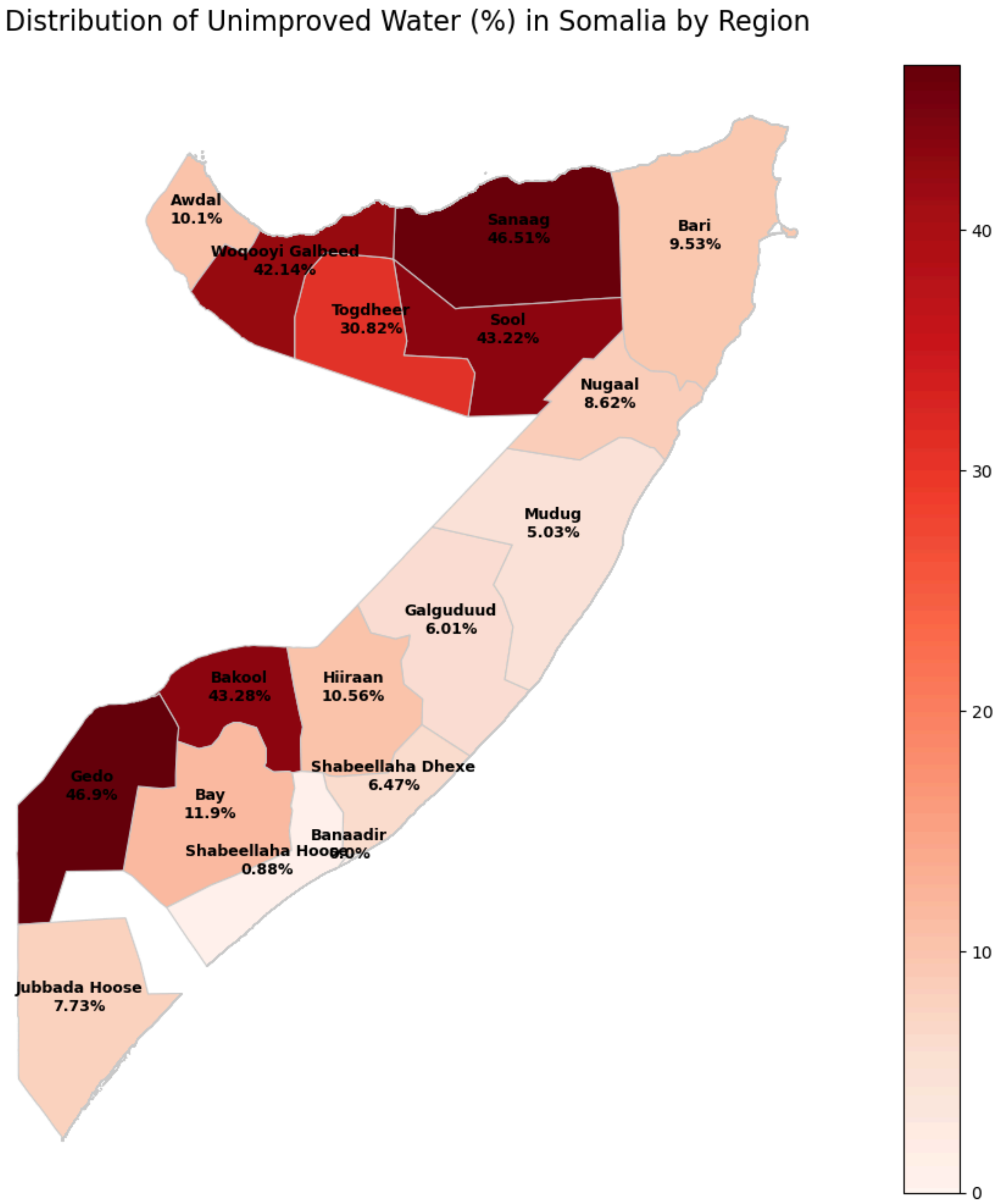

Distribution of Unimproved Water (%) in Somalia by Region
Awdal
10.1%
Woqooyi Galbeed
42.14%
Sanaag
46.51%
Bari
9.53%
Togdheer
30.82%
Sool
43.22%
Nugaal
8.62%
Mudug
5.03%
Galguduud
6.01%
Bakool
43.28%
Hiiraan
10.56%
Gedo
46.9%
Bay
11.9%
Shabeellaha Dhexe
6.47%
Banaadir
Shabeellaha Hoose
0.88%
Jubbada Hoose
7.73%
40
30
20
10
0


```
In [ ]:  1
```
