# Supplementary material for: Unimproved source of drinking water and its associated factors: Multivariable analysis of Somalia Integrated Household Budget Survey (SIHBS 2022)
Source: PLoS One. 2025 Oct 15;20(10):e0334606. doi: 10.1371/journal.pone.0334606 (PMC12527177; doi:10.1371/journal.pone.0334606)
Supplement: Attachment — Submitted filename: PONE.docx [file pone.0334606.s002.docx]

1. Rewrite the abstract in a proper structure (e.g., background, methods, results, conclusion).
2. Provide a more precise rationale for using a multivariable analysis.
3. The study uses cross-sectional data but occasionally provides causal conclusions. Causal conclusions cannot be validly drawn from such dataAuthors need to provide a clear description about reasons for selecting variables in the multivariable model.
4. Some AORs (e.g., for Lower Shabelle) have extremely wide CIs, suggesting sparse data or overfitting. This should be admitted.
5. The variable “food insecurity” shows a negative association with unimproved water use. It contradicts our expectation. Similar is the case with urban households. Authors can provide plausible reason behind it.
6. Authors can provide figures/maps to show regional variations.
7. Acknowledge the limitations of secondary data.
8. The use of terms like "community-factors factors" should be fixed throughout.
9. Several grammatical issues should be corrected.
